# Explorative analyses on the value of interim PET for prediction of response in pediatric and adolescent non-Hodgkin lymphoma patients

**DOI:** 10.1186/2191-219X-3-71

**Published:** 2013-10-18

**Authors:** Christian Furth, Ingo G Steffen, Anne S Erdrich, Patrick Hundsdoerfer, Juri Ruf, Günter Henze, Stefan Schönberger, Holger Amthauer, Hubertus Hautzel

**Affiliations:** 1 Department of Radiology and Nuclear Medicine, Medical School, Otto von Guericke University Magdeburg A.ö.R, Leipziger Strasse 44, Magdeburg 39120, Germany; 2 Department of Paediatric Oncology/Haematology, Campus Virchow, Charité-Universitätsmedizin Berlin, Berlin 13353, Germany; 3 Department of Pediatric Hematology and Oncology, University Children's Hospital Bonn, University of Bonn, Bonn 53113, Germany; 4 Department of Nuclear Medicine (KME) at the Research Center Juelich, Heinrich-Heine-University Duesseldorf, Juelich 40225, Germany

**Keywords:** FDG-PET, NHL, Response assessment, SUVmax, Therapy monitoring, Children

## Abstract

**Background:**

This study is to evaluate the predictive value of FDG-PET (PET) in pediatric and adolescent patients suffering from non-Hodgkin lymphoma (pNHL) in comparison to information provided by conventional imaging methods (CIM).

**Methods:**

Imaging was performed at baseline and at interim (after 2 cycles of chemotherapy). The response assessment in PET was carried out visually and semi-quantitatively, the latter one by use of percentage decrease in SUVmax from baseline to interim (*Δ*SUVmax). The PET-based results were compared to the findings by CIM. Progression-free survival (PFS) was analyzed using Kaplan-Meier curves (KM) and log-rank test.

**Results:**

The final study included 16 patients (mean follow-up time, 60.2 months (range, 4.0 to 85.7 months)). Relapse occurred in four patients. Visual PET compared to CIM revealed higher sensitivity (3/4 vs 1/4) and NPV (6/7 vs 10/13), and equal PPV (3/9 vs 1/3), but lower specificity (6/12 vs 10/12) and accuracy (9/16 vs 11/16). False-positive findings in PET at interim were predominantly observed in patients presenting bulky disease (5/6), whereas CIM was true-negative in all of these cases. KM analyses revealed no significant differences in 5-year PFS neither for CIM (76.9% vs 66.7%; *p* = 0.67) nor for visual PET (85.7% vs 66.7%; *p* = 0.34) nor for *Δ*SUVmax (88.9% vs 57.1%; *p* = 0.12).

**Conclusions:**

The predictive value of iPET in pediatric patients suffering from NHL was limited due to considerably high amount of false-positive findings, especially in patients suffering from bulky disease. However, due to our limited sample size, final conclusions cannot be drawn and, thus, call for further evaluation of PET in pNHL in larger and more homogenous patient series.

## Background

A deeper understanding of the biological behavior and the awareness that different subtypes of non-Hodgkin lymphoma (NHL) in pediatric and adolescent patients require different treatment strategies resulted in major improvements of cure rates ranging from more than 80% for lymphoblastic lymphoma up to 90% or even higher for mature B cell lymphoma [1]. However, the first-line treatment with curative intent is associated with considerable high toxicity due to dose-intense treatment strategies. On the other hand, in case of relapse, the chance for curative treatment is dramatically decreased due to unsatisfactory salvage regimen options. In order to facilitate a tailored, risk-adapted therapy, positron emission tomography (PET) using the tracer [^18^F]fluorodeoxyglucose (FDG) might contribute to identify patients with increased risk of relapse during or after treatment. Additionally, FDG-PET may help to distinguish patients with favorable outcome, who might benefit from a de-escalation of treatment intensity resulting in a reduction of treatment-related toxicity.

In adult NHL patients, FDG-PET negativity after completion of the first 2 cycles of chemotherapy was associated with a 2-year event free survival (EFS) of 82%, whereas 2-year EFS was only 43% in FDG-PET-positive patients [2]. Further on and more recently, in adult patients with diffuse large B cell lymphoma (DLBCL), Safar et al. were able to show that the percentaged decrease in "maximal standardized uptake value" (*Δ*SUVmax) between FDG-PET at baseline (bPET) prior to any anti-neoplastic treatment and FDG-PET at interim (iPET) can be used to effectively predict 3-year EFS [3]. The data on this topic are lacking in pediatric and adolescent patients suffering from NHL (pNHL).

This study presents prospective data on the potential of FDG-PET for early response assessment after completion of the first 2 cycles of chemotherapy in pNHL patients. Early response assessment was carried out (1) visually and (2) semi-quantitatively by analyses on *Δ*SUVmax between bPET and iPET, and (3) by a combination thereof. The findings in PET and conventional imaging modalities (CIM) were compared with regard to the prognostic value for prediction of progression-free survival (PFS).

## Methods

### Patients

The patients were prospectively recruited within the bounds of the PET2003 multi-center trial [4,5]. However, the present analyses are retrospective in nature. The exclusion criteria were life threatening impairment of organ function, pregnancy, diabetes mellitus or age younger than 1 year, respectively. Written informed consent was obtained from all the patients and/or parents. The study was carried out in accordance with the Declaration of Helsinki and the principles of good clinical practice. The institutional review board (Charité - Universitätsmedizin Berlin) approved the study protocol. The approval was granted by the German Federal Office on Radiation Protection (Bundesamt für Strahlenschutz) as well as by the corresponding local authorities. Eighteen pediatric and adolescent patients (female, 5; male, 13; mean age, 13.5 years (3.7 to 23.2 years)) with NHL (Burkitt/Burkitt-like, *n* = 7; DLBCL, *n* = 5; anaplastic large cell lymphoma (ALCL), *n* = 3; T lymphoblastic, *n* = 2; follicular (WHO grade 2), *n* = 1) were analyzed (Table 1). Fifteen patients with newly diagnosed NHL were included for analyses, whereas three patients (patient 8, patient 17, and patient 18) were included after diagnosis of first relapse of the disease. All patients were treated according to the appropriate therapy optimization protocol (TOP) [6-8]. With respect to the treatment of the recurrent disease, the ALCL-relapse TOP [9] was used.

**Table 1:** Patients' data and clinical characteristics

**Characteristics**	**Patients (*n *= 18)**
Gender (*n*)	
Female	5
Male	13
Age (years)	
Median	13.5
Range	3.7 to 23.2
Histology (*n*)	
Burkitt/Burkitt-like	7
DLBCL	5
ALCL	3
Lymphoblastic	2
Follicular (WHO grade 2)	1
Involvement (*n*)	
Nodal only	6
Nodal and extra nodal	10
Bone marrow	5
Follow-up (months)	
Mean	60.2
Range	4.0 to 85.7

ALCL, anaplastic large cell lymphoma; DLBCL, diffuse large B cell lymphoma; *n*, count; WHO, World Health Organization.

The treatment of more than 95% of children with malignant disease occurs according to TOPs. The patients with lymphoblastic lymphoma, B-NHL, ALCL, and relapse of ALCL were treated according to the TOPs Euro-LB 02, B-NHL BFM 2004, ALCL99, and ALCL-relapse, respectively. All TOPs include intensive multi-drug chemotherapy for 6 to 9 months. In patients with lymphoblastic lymphoma, intensive polychemotherapy is followed by oral maintenance chemotherapy for a total therapy duration of 2 years.

The mean follow-up was 60.2 months (range, 4.0 85.7 months). The time point of analysis was 8 February 2012. Due to violation against PET imaging protocol (prolonged uptake time at baseline PET scan), two patients had to be excluded from the analyses (patient 4 and patient 6) (Table 2).

**Table 2:** Response assessment by conventional imaging modalities and positron emission tomography at interim time point

**Patient no.**	**Stage**	**Bulky disease**	**Histopathological subtype**	**Localization of leading lesion**	**Response assessment at interim**			**Follow-up**
					**iCIM**	**iPET**	**ΔSUVmax (%)**	
1	III	B	lymphoblastic	Nodal	CRu	+	88.1	CR
2	II	-	DLBCL	Nodal	PR	-	92.2	CR
3	IV	-	DLBCL	Bone/bone marrow	SD	-	94.5	CR
4	II	-	Follicular grade 2	Nodal	CR	+	*	CR
5	IV	B	DLBCL	Nodal	CRu	+	60.8	CR
6	IV	B	DLBCL	Nodal	CRu	+	*	CR
7	II	-	Burkitt	Nodal	CR	-	97.2	CR
8	III	-	ALCL	Nodal	CR	+	47.8	Relapse
9	I	B	Burkitt	Nodal	CRu	+	91.2	CR
10	IV	B	Burkitt	Nodal	CR	-	95.2	CR
11	IV	-	Burkitt	Nodal	CRu	+	90.5	Relapse
12	II	B	ALCL	Nodal	CRu	-	95.8	CR
13	IV	-	Lymphoblastic	Nodal	PR	+	88.5	Relapse
14	III	-	ALCL	Nodal	CRu	+	34.1	CR
15	III	B	Burkitt	Extra nodal (stomach)	CR	+	50.7	CR
16	IV	B	Burkitt	Nodal	CR	+	92.5	CR
17	I	-	DLBCL	Nodal	CR	-	95.8	Relapse
18	IV	-	Burkitt	Nodal	CR	-	93.5	CR

-, negative; +, positive; *, censored from analyses due to violation against imaging protocol (prolonged tracer uptake time at time point of staging, *n* = 2); B, bulky disease present; CIM, conventional imaging modalities; CR, complete response, CRu, complete response unconfirmed; PET, positron emission tomography; PR, partial response; SD, stable disease.

### CIM: acquisition and analysis

CIM according to TOPs consisted of contrast-enhanced magnetic resonance imaging (MRI) of neck, abdomen, and pelvis; ultrasounds of all the lymph node regions; as well as a contrast-enhanced thoracic computed tomography (CECT). In case of clinical suspicion, additional examinations by CIM were performed (e.g., lower extremity, cranial/spinal MRI). The specifications of CIM devices used and technical requirements needed are published elsewhere [10]. The data of CIM were reviewed by two experienced radiologists in a consensus reading, blinded to the results of PET and clinical follow-up data using a dedicated work station (AdvantageWindows 4.1, GE Medical Systems, Milwaukee, IL, USA). The reading of CIM for assessment of response status was done in accordance to the criteria defined by an international workshop [11], including complete response (CR), CR unconfirmed (CRu), partial response (PR), stable disease (SD), and progression of disease (PD). The patients with CR or CRu at interim were judged to be iCIM-negative, whereas the patients stated to have PR, SD, or PD were categorized to be iCIM-positive.

### PET: acquisition and reconstruction analysis

FDG-PET scanning was performed for staging prior to any administration of anti-neoplastic treatment (bPET) and after completion of the first 2 cycles of chemotherapy (iPET), with a minimum time interval between the preceding chemotherapy and iPET of 14 days. Whole-body FDG-PET examinations were performed according to the recommendations of the "European Association of Nuclear Medicine" guidelines [12] (activity at bPET: mean, 222 MBq (standard deviation (SDev), ±71.7 MBq); activity at iPET: mean, 250 MBq (SDev, ±75.3 MBq)). The PET protocol consisted of an 8-hour fasting period, followed by confirmatory blood sugar testing to ensure that the glucose values were within the normal range (reference, <6.1 mmol/L).

The scanners used were dedicated full-ring PET systems (ECAT EXACT 921, 47, Siemens, Erlangen, Germany; ECAT EXACT HR+, Siemens/CTI, Knoxville, TN, USA) or a hybrid PET/CT (Biograph16, Siemens, Erlangen, Germany). The children were consistently examined with the same device at interim as used for staging. All the PET scans were performed in two-dimensional mode (six to eight bed positions with 8 min of emission and 4 min of transmission) (FDG uptake time at bPET: mean, 88 min (SDev, ±19.1 min); FDG uptake time at iPET: mean, 76 min (SDev, ±20.4 min)). In case of a clinical suspicion, additional PET imaging was performed (e.g., lower extremity). Regarding the differences in the scanner performance within this multi-center trial, appropriate scanner calibrations had been performed to keep discrepancies at minimum level. The PET data were reconstructed using ordered subset expectation maximization with 4 iterations and 16 subsets, using a matrix of 128 × 128 for the stand-alone PET scanner and a matrix of 256 × 256 for the PET/CT device. The emission data were corrected for decay, dead time, scatter, and random coincidences. The parameters for the PET acquisition were in accordance with the recommendations given by Boellaard et al. [13].

### PET: visual analysis

For visual evaluation, maximum intensity whole-body projections as well as coronal, axial, and sagittal slices were reconstructed from the PET datasets (e.soft4.0, Leonardo workstation, Siemens Medical Solutions, Erlangen, Germany). Regarding the performed FDG-PET/CT studies, the acquired functional and morphological datasets were read separately. Thus, each method was evaluated independently without prior knowledge of the PET or CIM findings, respectively. The PET data were evaluated in consensus by two experienced nuclear medicine specialists blinded to the results of CIM and clinical data. The PET reading was carried out in accordance with criteria defined by the International Harmonization Project in lymphoma (IHP) [14]. The PET scans were assessed visually and judged positive in case of detection of focally increased uptake above the surrounding tissue or increased uptake as compared to mediastinal blood pool activity which was used as region of reference in terms of FDG accumulation. However, mild and diffusely increased FDG uptake with an intensity lower than or equal to that of mediastinal blood pool structures was considered to be PET-negative.

### PET: semi-quantitative analysis

For semi-quantitative analyses, attenuation-corrected PET datasets were used only. Lesions with pathologically increased FDG uptake obtained in the visual response assessment were determined by SUVmax using a "region of interest" (ROI) feature of a dedicated software tool (rover® v2.0.31, ABX GmbH, Radeberg, Germany). Within all the lesions measured in bPET, the highest SUVmax (SUVmax bPET) was documented and then re-evaluated in iPET (SUVmax iPET) at that same site in the respective patient. In case of a complete metabolic response at iPET as assessed by visual means, a minimized standard mask consisting of three voxels was placed in the center of the initially affected lymphoma site. The relative SUVmax differences were calculated as *Δ*SUVmax = (SUVmax bPET - SUVmax iPET) / SUVmax bPET × 100.

### PET: analyses on combined assessment

Patients were defined to be positive in combined assessment if iPET was visually positive and *Δ*SUVmax was less than or equal to the corresponding cutoff in receiver operator characteristics (ROC) analyses. All the other combinations were stated to be negative in combined iPET assessment.

### Standard of reference

To establish a standard of reference, the results of CIM and PET were finally verified by an interdisciplinary tumor board. For verification of the lesion status, all staging and follow-up examinations, histopathology of biopsies, and clinical data including the serial follow-up examinations were used. All patients underwent regular follow-up investigations, including physical examinations, blood tests, chest X-ray, and ultrasound quarterly during the first year, half yearly during the second year, and once a year thereafter. Suspicion of relapse was confirmed by biopsy and histopathological examination. Progression-free survival (PFS) was defined as the time from enrollment in PET2003 trial to first progression, relapse, and either death, whatever the cause, or last follow-up.

### Statistics

The data analyses were carried out using the software R version 2.14.0 (R Foundation for Statistical Computing, Vienna, Austria; http://www.R-project.org). The clinical characteristics and quantitative data for the obtained SUVs are expressed as median ± SDev, unless otherwise specified. Sensitivity, specificity, positive predictive value (PPV), and negative predictive value (NPV), as well as accuracy, were calculated using standard formulas. The differences of diagnostic parameters were tested according to the method of Bennett [15]. A ROC analysis was performed to analyze the association of *Δ*SUVmax and the recurrence of disease. The optimal ROC threshold was defined by the point with the minimal distance to 100% sensitivity and 100% specificity. The PFS was analyzed using Kaplan-Meier curves and log-rank test. All tests were two sided, and a *p* value less than 0.05 was referred to be significant.

## Results and discussion

Two patients (patient 4 and patient 6) had to be excluded from the analyses due to violation against PET imaging protocol (prolonged uptake time at bPET) (Table 2). In four of the remaining 16 patients, relapse of disease occurred (patient 11, 3.1 months; patient 13, 7.4 months; patient 8, 24.3 months; patient 17, 25.7 months). Two patients died during follow-up due to progression of disease (patient 11, 4.0 months; patient 13, 28.8 months) (Table 1). The lesions used for response assessment at interim were localized at nodal sites in 14 patients and in 2 patients at extra nodal sites (Table 2).

### Visual response assessment: iCIM versus iPET

Concerning visual assessment, iCIM and iPET revealed concordant findings in six patients (TP, *n* = 1; TN, *n* = 4; FN, *n* = 1). In two patients, iPET was TP, whereas iCIM was FN; vice versa, in two patients, iPET was TN, and iCIM revealed FP findings. In the remaining six cases, iCIM was TN, whereas iPET revealed FP findings.

iPET revealed higher sensitivity (3/4) compared to iCIM (1/4) and higher NPV (iPET, 85.7% (6/7) vs iCIM, 76.9% (10/13)). Higher specificity was observed using iCIM (iCIM, 83.3% (10/12) vs iPET, 50.0% (6/12)). Comparable values were obtained for PPV (iCIM, 33.3% (1/3) vs iPET, 33.3% (3/9)). In total, accuracy was higher in iCIM (11/16, 68.8%) when compared to iPET (9/16, 56.2%). A comprehensive overview of the diagnostic values obtained is given in Table 3. No significant differences were observed (sensitivity, *p* = 0.157; specificity, *p* = 0.157; NPV, *p* = 0.480; PPV, *p* = 1).

**Table 3:** Overview of diagnostic values obtained by tested assessment approaches

	**Assessment approach**		
	**iCIM**	**iPET**	**ΔSUVmax**
TP (*n*)	1	3	3
FP (*n*)	2	6	4
TN (*n*)	10	6	8
FN (*n*)	3	1	1
Sensitivity, % (CI)	25.0	75.0	75.0
	(1.3-70.0)	(30.1-98.7)	(30.1-98.7)
Specificity, % (CI)	83.3	50.0	66.7
	(55.2-95.3)	(25.4-74.6)	(39.1-86.2)
PPV, % (CI)	33.3	33.3	42.9
	(1.7-79.2)	(12.1-64.6)	(15.8-75.0)
NPV, % (CI)	76.9	85.7	88.9
	(49.7-91.8)	(48.7-99.3)	(56.5-99.4)
Accuracy, % (CI)	68.8	56.2	68.8
	(44.4-85.8)	(33.2-76.9)	(44.4-85.8)

*Δ*, percentaged decrease of SUVmax between staging and restaging; CI, confidence interval; FN, false-negative; FP, false-positive; iCIM, conventional imaging modalities performed at interim; iPET, positron emission tomography performed at interim; *n*, count; NPV, negative predictive value; PPV, positive predictive value; SUVmax, maximum standardized uptake value; TN, true-negative; TP, true-positive.

### Semi-quantitative analyses of FDG uptake reduction induced by the first 2 cycles of polychemotherapy

Median SUVmax at initial staging was 17.1 (range, 8.5 to 49.7). After 2 cycles of polychemotherapy, a median SUVmax reduction of 91.7% (range, 34.1% to 97.2%) was observed, corresponding to a median SUVmax of 2.0 (range, 0.7 to 10.6) at interim. The ROC analysis of SUVmax reduction at interim for identification of relapse patients showed an AUC of 0.58 (*p* = 0.684) with a corresponding optimal cutoff value at 90.5%. Three of the seven patients with SUVmax reduction ≤90.5% suffered relapse of disease. Sensitivity and specificity for the prediction of relapse in *Δ* analyses were 75.0% (3/4) and 66.7% (8/12), respectively (PPV, 42.9% (3/7); NPV, 88.9% (8/9); accuracy, 68.8% (11/16)). No statistical differences were seen when the diagnostic values of the *Δ*SUVmax analyses were compared to the diagnostic values of iCIM interpretation (sensitivity, *p* = 0.157; specificity, *p* = 0.414; PPV, *p* = 0.732; NPV, *p* = 0.294).

### Combined response assessment by FDG uptake reduction and visual response assessment by iPET

The combined approach showed no improvement as all the patients judged to be negative in visual iPET assessment showed a *Δ*SUVmax ≥90.5% (ROC cutoff).

### Prediction of progression-free survival

**Figure 1:**
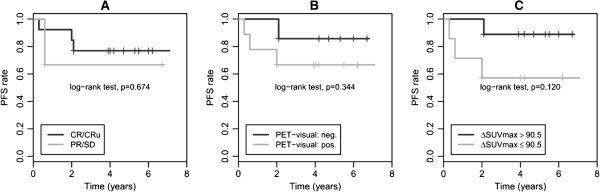
**Kaplan-Meier curves for response assessment by conventional imaging modalities and positron emission tomography.** Progression-free survival according to the information provided by the response assessment using **(A)** conventional imaging modalities in accordance to the recommendations given by Cheson et al. [11], **(B)** visual response assessment of interim PET using the recommendations given by Juweid et al. on behalf of the IHP [14], and **(C)** relative decrease in SUVmax between baseline and interim PET. PFS, progression-free survival; CR, complete response; CRu, complete response unconfirmed; PR, partial response; SD, stable disease; *Δ*SUVmax, decrease in maximum standardized uptake value between staging and interim PET; neg., negative; pos., positive.

The Kaplan-Meier curves for 5-year PFS are given in Figure 1 (Figure 1A,B,C) and showed no significant differences in PFS, neither for CIM (76.9% vs 66.7%; *p* = 0.67) nor for visual iPET analyses (85.7% vs 66.7%; *p* = 0.34) nor for *Δ*SUVmax analyses (88.9% vs 57.1%; *p* = 0.12).

The role of interim PET in NHL is discussed controversially with respect to the response to therapy assessment [16-20]. Visual response assessment in pNHL using dedicated, well-defined response criteria was limited to the concordant detection of patients suffering early relapse, whereas patients with late relapse were not reliably detectable in a blinded multi-center read [21]. In adult patients suffering from NHL (i.e., DLBCL), the results suggest that the use of SUVmax in addition to visual analyses substantially improves the prognostic value of iPET [22].

In our pediatric population, the use of semi-quantitative response assessment by means of *Δ*SUVmax analyses indicated a slightly improved sensitivity as well as a slightly improved NPV for the detection of patients with subsequent relapse. However, CIM was superior to *Δ*SUVmax concerning specificity, whereas PPV was poor for both approaches. The Kaplan-Meier curves showed no significant differences concerning the prediction of PFS neither for CIM nor for visual iPET assessment nor for *Δ*SUVmax analyses.

The data addressing the role of interim or end of treatment PET in pNHL are rare. Regarding the prediction of response to therapy, Edeline et al. [23] reported that information gathered from end of treatment PET (visual assessment) in ten pediatric patients suffering from NHL did not provide further information to accurately predict outcome. In their series, only one out of five PET-positive pNHL patients showed a relapse, whereas four out of five PET-negative patients remained in remission. In contrast, Depas et al. [24] found an excellent specificity for PET when performed during treatment (range, PET after 2 to 3 cycles of chemotherapy) as there were no false-positives in their analyses. However, PET failed to identify the recurrence of disease in all three patients suffering relapse, suggesting a more specific but not sensitive read out. Similar observations were done by Mody et al. [25], as PET was not suitable to detect recurrence of disease in the CNS. However, close location of CNS lymphoma to areas of physiologically high uptake is a well-known pitfall in PET imaging. A more recent study investigating 34 pNHL patients found a PPV of 41.2% (7/17 patients) and a NPV of 85.7% for interim PET/CT analyses [26]. The poor PPV (33.3%, 3/9 patients) and high NPV (85.7%, 6/7) for the visual iPET response assessment in our study is comparable to the results presented by Bakhshi et al. [26]. However, more important and similar to our results, they also found no significant discordance in prediction of PFS when findings of CECT and PET/CT were compared to the true outcome of each patient (CECT, *p* = 0.18; PET/CT, *p* = 0.083) [26].

This particular observation is in contrast to the data on iPET in adults suffering from DLBCL reported recently. Safar et al. [3] reported that 3-year PFS in 112 adults suffering from DLBCL was significantly higher in iPET-negative patients (84%) compared to those in iPET-positive patients (47%, *p* < 0.0001) using visual response assessment. However, the results from the LNH2007-3B trial [22] showed that the visual response assessment by iPET was not suitable to significantly improve the prediction of 2-year PFS (iPET-negative, 77%; iPET-positive, 73%; *p* = 0.586), whereas *Δ*SUVmax analyses showed significant improvement of 2-year PFS (iPET-negative *Δ*SUVmax ≤66%, 57% vs iPET-positive *Δ*SUVmax >66%, 77%; *p* = 0.028) [22]. The latter one was confirmed by Safar et al. for the prediction of 3-year PFS using the same cutoff in *Δ*SUVmax analyses (iPET-negative *Δ*SUVmax ≤66%, 77% vs iPET-positive *Δ*SUVmax >66%, 38%; *p* = 0.002) [3]. However, in our study, the use of *Δ*SUVmax analyses with a threshold of 90.5% as revealed by ROC analysis was not suitable to significantly predict PFS (*p* = 0.120). Applying the *Δ*SUVmax cutoff revealed by Casasnovas et al. [22] and Safar et al. [3] (both iPET-negative *Δ*SUVmax ≤66%) on the present patient series, no significant differences in 5-year PFS were observed too (both groups 75%, data not shown). Overall, the performance of *Δ*SUVmax analyses in our study was disappointing compared to the results in adults. As ROC analysis showed no significant discrimination concerning the prediction of response, the Kaplan-Meier curves have to be interpreted with caution.

The present study revealed a high amount of false-positive findings in PET, all being true-negative in CIM. Due to not specific nature of the tracer used, there is a high likelihood for false-positive PET findings during treatment (e.g., brown fatty tissue, fat necrosis, inflammation, reactive lymph nodes, thymus rebound, and diffuse bone marrow uptake after administration of hematopoietic growth factors) [27-29]. A high number of false-positive findings (26/38) has also been reported by Moskowitz et al. in a prospective series [19]. Serial histology confirmed in only 5 out of 38 patients with viable lymphoma cells. In the remaining 33 patients, progression of the disease occurred in seven patients [19].

In the present study, the false-positive findings in iPET were predominantly observed in patients presenting bulky disease (visual iPET, 5/6 patients). The iPET false-positive findings in these patients may be explained by a stromal reaction due to influx of inflammatory cells (i.e., macrophages) as it has been demonstrated in a mouse model [30]. The uptake due to non-lymphoma-related cells might be associated with the extension of the bulky lesion at baseline. These findings may become important in reading and interpreting of iPET in patients presenting bulky disease at baseline.

**Figure 2:**
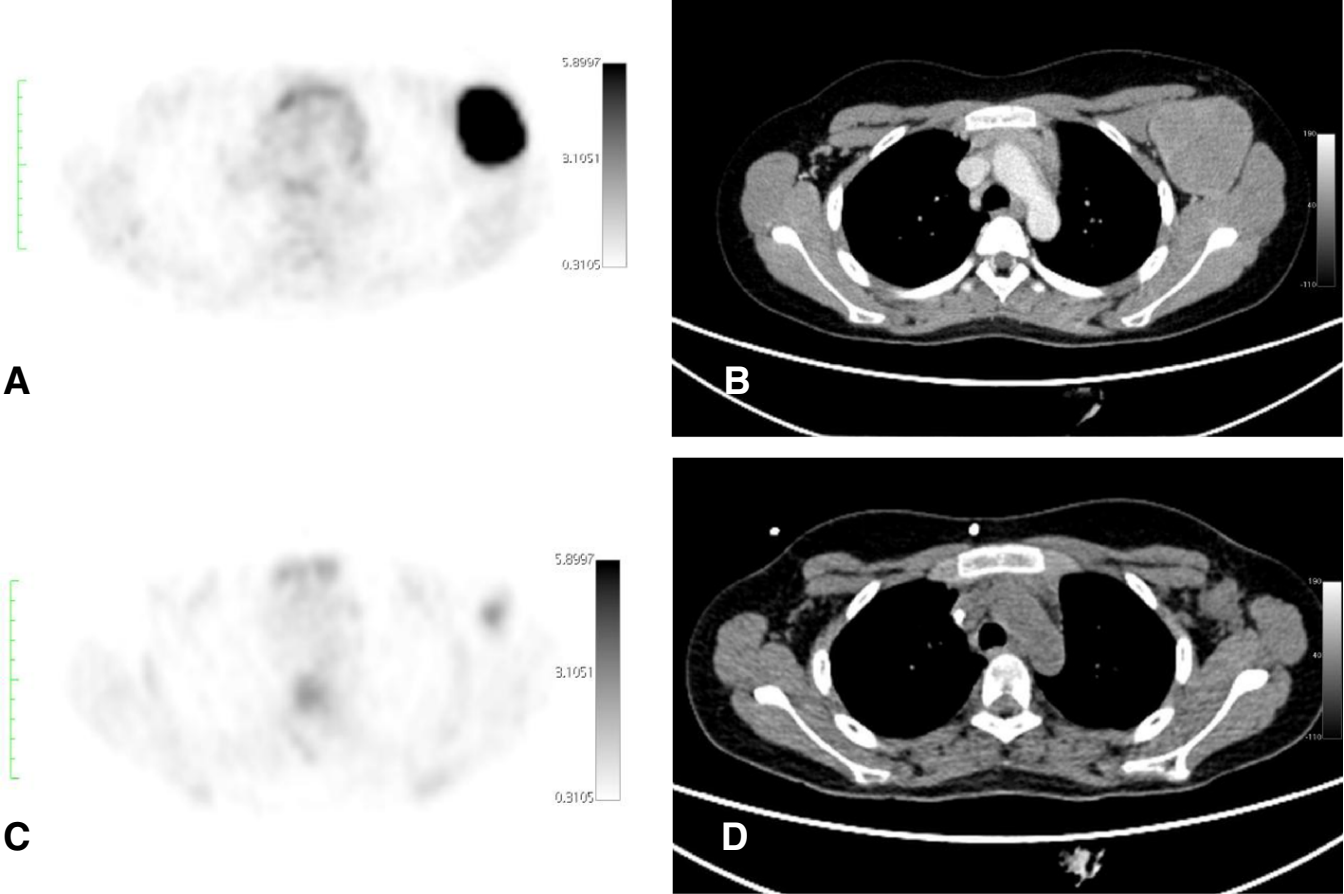
**Twelve-year-old boy suffering from Burkitt lymphoma (Table**2**, patient 9).** The lymphoma was localized in the left axilla **(A**, **B)**. SUVmax at baseline was 30.9 and decreased to 2.7 at interim. Visual iPET assessment was judged to be iPET-positive **(C)**, whereas *Δ*SUVmax analyses indicated a sufficient decrease (*Δ*SUVmax, 91.2%). CIM **(B**, **D)** confirmed a complete response unconfirmed at interim. Histological examination of the visual iPET-positive finding ruled out viable lymphoma cells. The patient is still in remission (clinical follow-up, 66 months).

**Figure 3:**
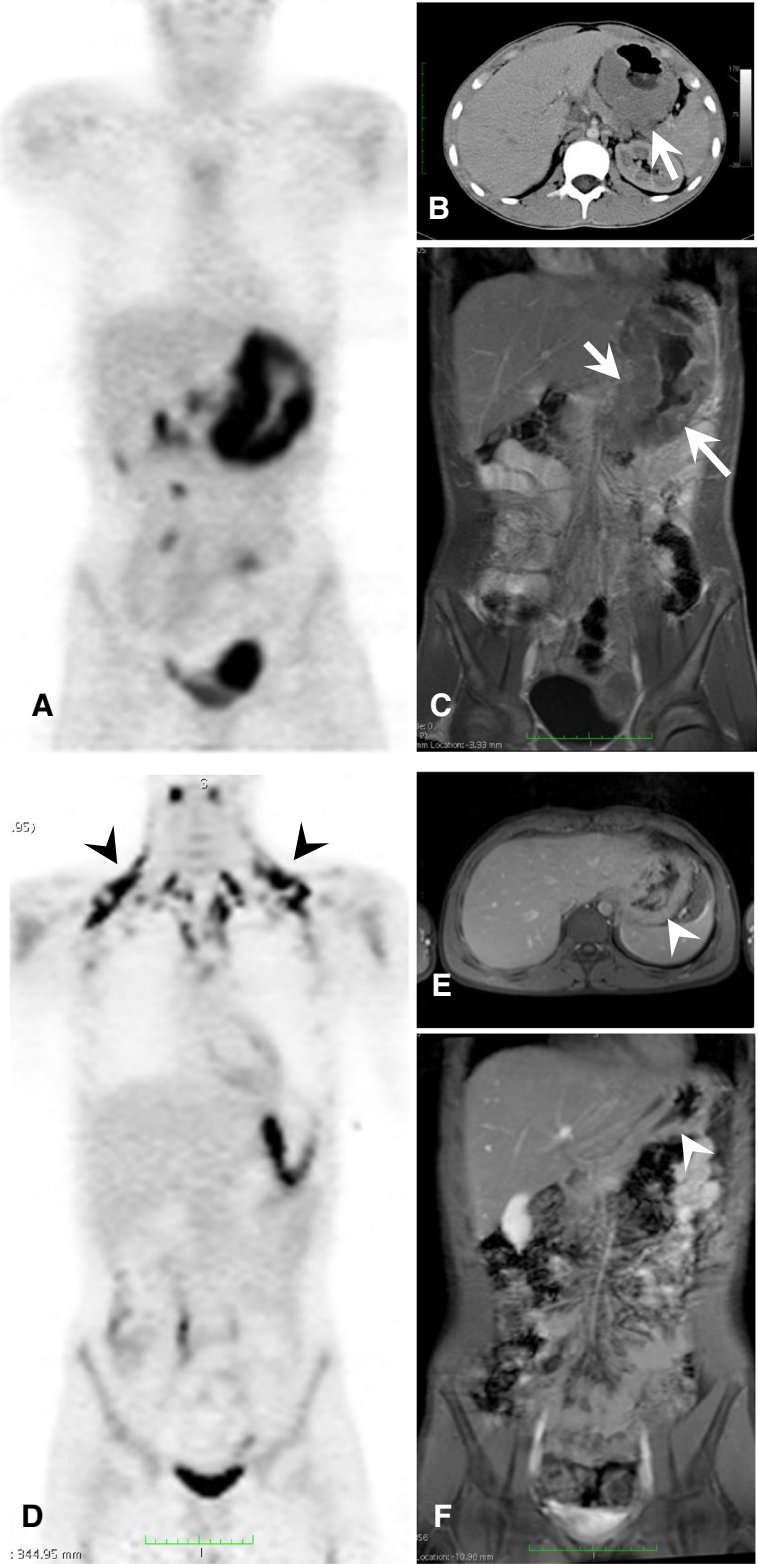
**Fourteen-year-old boy suffering from Burkitt-lymphoma (Table**2**, patient 15).** The lymphoma was primarily localized in the stomach **(A**, **B**, **C)**. Initial SUVmax was 21.5 and decreased to 10.6 at interim time point (**A** and **D**; *Δ*SUVmax, 50.7%). Also, in the visual iPET analysis **(D)**, the patient was judged to be positive (please note activation of brown adipose tissue at interim marked with black arrow heads **(D)**. Using CIM, a decrease of 100% between baseline (arrows in **B** and **C**) and interim time points was shown (arrow heads in **E** and **F**). No further measurable disease was detected by CIM. Thus, a complete response was indicated by CIM. The patient is in ongoing remission. Therefore, both *Δ*SUVmax and visual iPET analyses were false-positive when compared to true outcome, whereas CIM was true-negative.

**Figure 4:**
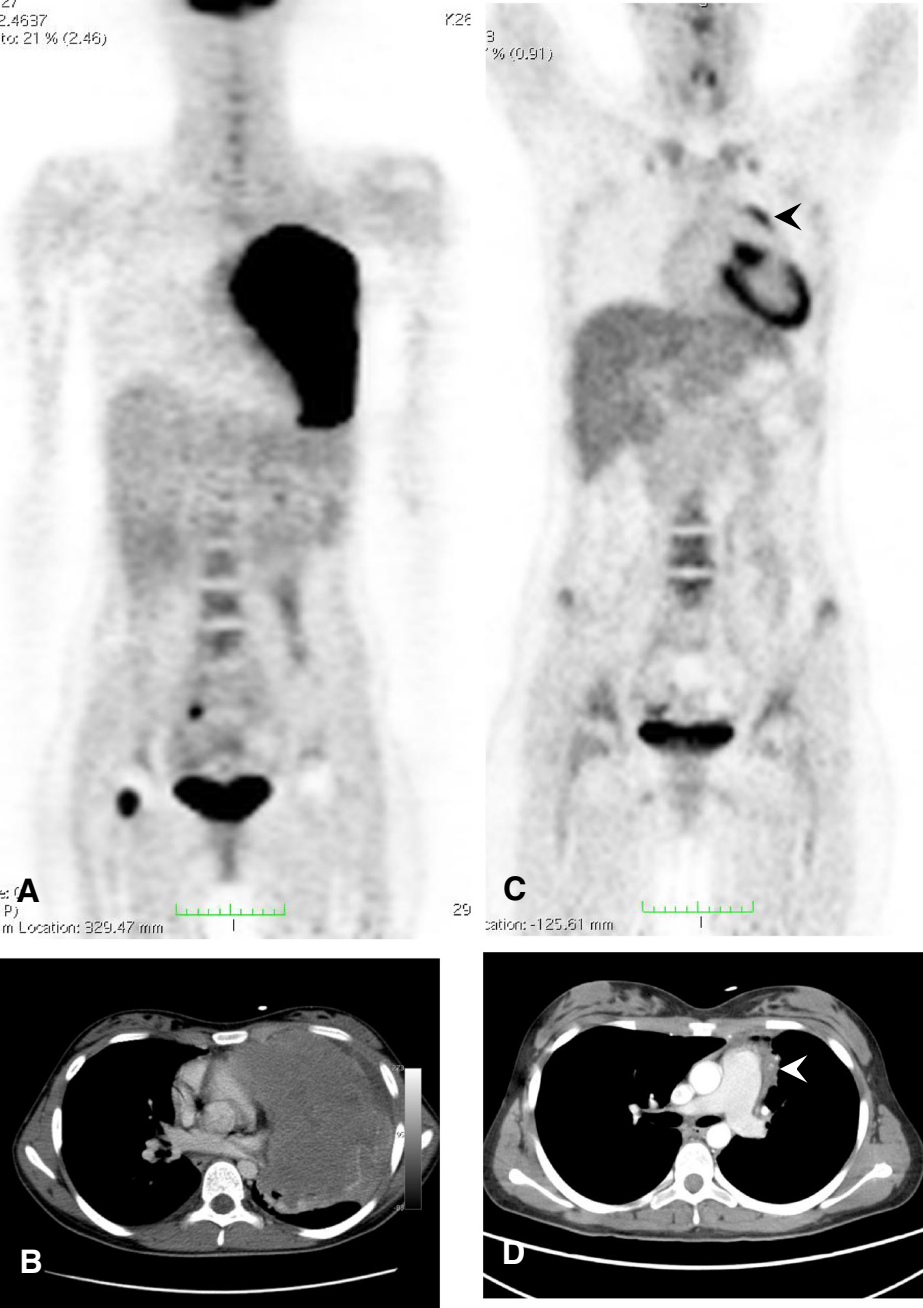
**Sixteen-year-old girl suffering from a diffuse large B cell lymphoma (Table**2**, patient 5).** The lymphoma was primary localized in the left lung and pleura **(A**, **B)**. Initial SUVmax was 10.7 and decreased to 4.2 at interim (**A** and **C**; visual iPET-positive, *Δ*SUVmax 60.7%). Thus, PET approaches suggested residual disease at interim. CIM findings at interim showed massive reduction in size (arrow head, **D**) and disappearance of all other foci corresponding to a complete response unconfirmed. The patient is in ongoing remission (CIM, true-negative; visual iPET, false-positive; *Δ*SUVmax, false-positive).

Due to ethical reasons, serial histological confirmation of iPET-positive findings in children is beyond ethical reasons; however, it was performed in one patient in our series (Table 2; patient 9, Figure 2). The histological verification of his iPET-positive finding showed a reactive infiltrate of macrophages, particularly foamy cells and a few lymphoid aggregates, but no viable lymphoma cells [21]. Further on, Figures 3 and 4 illustrate two exemplary cases for iPET-positive findings (Table 2; patient 5 and patient 15) which turned out to be false-positive (Figures 3 and 4). In both cases, increased tracer uptake within the residual at interim may be attributed to the influx of inflammatory cells.

Nevertheless, as iPET is acquired after 2 cycles of chemotherapy while that particular PET result is compared to the result after the entire treatment, the possibility of a "true-positive iPET signal" (generated by at that time still viable lymphoma tissue that was only later successfully eradicated by subsequent treatment) cannot be safely excluded.

Several limitations of our study have to be addressed: (1) The number of patients included in our analyses is too small, and the variety of histological subtypes is too big to draw final conclusions; thus, further prospective investigations in prospectively enrolled series with proper sample sizes are needed. (2) Use of different PET devices may confound the results of the presented study as it may contribute to SUV variability. However, this problem might be overcome by close adherence to standardized protocols as initiated by the European Association of Nuclear Medicine (i.e., EARL FDG-PET/CT Accreditation) [13]. (3) Sub-summation of various stages (e.g., early, intermediate, and advanced) hamper an unbiased comparison to data in adults as that data were obtained in more homogeneous series. However, homogeneous pediatric patient series with sufficient sample sizes are difficult to obtain even in multi-center trials. (4) Lastly, the optimal time point of PET imaging in pNHL with positive findings minimally falsified by inflammatory or reactive reactions still has to be determined.

## Conclusions

In our explorative analyses, the predictive value of PET for response assessment at interim in pediatric patients suffering from NHL was limited. Whereas the underperformance of visual iPET assessment was expectable, especially the poor performance of the *Δ*SUVmax approach was a disappointing finding. Especially in patients with bulky disease, the PET approaches mainly failed to predict the outcome. However, due to the small number of included patients, final conclusions cannot be drawn and, thus, call for further evaluation of PET in pNHL in larger and more homogenous patient series.

## Competing interests

The authors declare that they have no competing interests.

## Authors' contributions

CF and HH collected the data and drafted the manuscript. IGS drafted the manuscript. ASE revised the manuscript critically for important intellectual content. PH collected the data, initiated the conception and design of the study, and revised the manuscript critically for important intellectual content. JR collected the data and revised the manuscript critically for important intellectual content. GH contributed to the acquisition of funding and was part of the conception and design of the study. SS collected data and revised the manuscript critically for important intellectual content. HA was part of the conception and design of the study. All authors read and approved the final manuscript.
